# Dendritic Cell-Cytokine-Induced Killer Cells Co-Loaded with WT1/MUC1/Poly(I:C) Enhance Antitumor Immune Responses In Vitro and In Vivo

**DOI:** 10.3390/biom15101356

**Published:** 2025-09-24

**Authors:** Huimin Liu, Chenlong Wang, Hongtao Chang, Liangliang Dong, Guoqing Yang, Cailing Tong, Lin Mao

**Affiliations:** 1 College of Life Sciences, Henan Agricultural University, Zhengzhou 450046, China; liuhuimin@henau.edu.cn (H.L.); chenlw@gamil.com (C.W.); dongll@gmail.com (L.D.); gqyang@henau.edu.cn (G.Y.); 2 College of Veterinary Medicine, Henan Agricultural University, Zhengzhou 450046, China; changht@henau.edu.cn; 3 Biocomer Technology Co., Ltd., Xiamen 361011, China

**Keywords:** MT1, MUC1, poly(I:C), DC-CIK cells, antitumor

## Abstract

Dendritic cell-cytokine-induced killer (DC-CIK) therapy faces limitations due to antigenic heterogeneity and suboptimal immune activation. In this study, we developed a multi-antigen-loaded DC-CIK (Ag-DC-CIK) system that co-targets Wilms’ tumor 1 (WT1), mucin-1 (MUC1), and the TLR3 agonist poly(I:C) to improve therapeutic outcomes. Utilizing umbilical cord blood-derived DC and CIK cells, we demonstrated that Ag-DC-CIK significantly enhanced cytotoxicity, as evidenced by the lactate dehydrogenase (LDH) assay, and increased apoptosis induction, indicated by elevated Bax and reduced Bcl-2 expression, in various tumor cell lines (HeLa, HCT116, MKN45) and organoids generated from a gastric cancer patient. Furthermore, Ag-DC-CIK effectively suppressed tumor cell migration and reduced the viability of the organoid. In MKN45 xenograft models, Ag-DC-CIK treatment inhibited tumor growth without inducing systemic toxicity, as shown by decreased Ki67 cell proliferation. This tripartite strategy synergistically enhances DC-CIK therapy by expanding antigen recognition and augmenting immune responses, presenting a promising translational approach for the treatment of gastric cancer.

## 1. Introduction

Cancer immunotherapy has revolutionized oncology by harnessing the immune system to selectively target cancerous cells, thereby reducing systemic toxicity in comparison to conventional therapies. Among emerging strategies, dendritic cell-cytokine-induced killer (DC-CIK) cell therapy integrates antigen-presenting dendritic cells (DCs) with cytokine-activated CIKs, effectively combining immune activation with direct tumor cytotoxicity [1,2]. Although DC-CIK therapy has shown clinical promise in hematologic malignancies and solid tumors (e.g., non-small-cell lung cancer and gastric carcinoma) [3,4], its efficacy remains constrained by several limitations [5]. A primary limitation of conventional DC-CIK methodologies is their reliance on tumor lysates or single-antigen loading, which inadequately address tumor heterogeneity and result in inconsistent immune responses due to variable antigen presentation and immune evasion mechanisms.

To address these challenges, recent research has concentrated on tumor-associated antigens with extensive applicability across cancer types. Notably, Wilms’ tumor 1 (WT1) and mucin-1 (MUC1) have been identified as high-priority targets due to their overexpression in multiple malignancies and their pivotal roles in oncogenesis. WT1, a zinc finger transcription factor, exhibits dual roles as both a tumor suppressor and oncogene [6,7,8,9]. Early-phase clinical trials investigating WT1-targeted immunotherapy have reported safety and preliminary efficacy [10,11]. Similarly, MUC1 (CD227), a transmembrane glycoprotein commonly overexpressed in breast and gastric cancers, facilitates immune evasion by disrupting cell adhesion and promoting immunosuppressive signaling pathways [12,13,14]. Poly(I:C), a Toll-like receptor 3 (TLR3) agonist, has been explored as an adjuvant in cancer immunotherapy [15]. In a study employing B16-OVA melanoma, Panc-OVA pancreatic, and TRAMP-C1 prostate cancer mouse tumor models, the simultaneous administration of an ISCOMATRIX vaccine with poly(I:C) and a TLR9 agonist resulted in a significant reduction in tumor growth across all models [16]. The inclusion of ISCOMATRIX in the formulation was essential for the vaccine’s therapeutic efficacy, as this combination elicited a robust and multifunctional CD8^+^ T cell response. These preliminary findings have stimulated further research into the application of poly(I:C) in combination with other immunotherapeutic agents to enhance the anti-tumor immune response. However, the potential synergistic application of WT1, MUC1, and poly(I:C) in DC-CIK therapy remains unexplored.

In this study, we have developed a multi-antigen-loaded DC-CIK (Ag-DC-CIK) system to effectively address tumor heterogeneity and immunosuppression. Through in vitro assays, patient-derived organoids, and xenograft models, we demonstrate that the Ag-DC-CIK system induces broad-spectrum cytotoxicity, promotes apoptosis induction, and inhibits migration. Our findings offer a framework for precision immunotherapy in gastric cancer and potentially other malignancies.

## 2. Materials and Methods

### 2.1. Cell Lines and Reagents

All cell lines (HeLa, HCT116, THP-1, MKN45, KatoIII, and N87) were obtained from ATCC and maintained in RPMI-1640 (Gibco, Ottawa, ON, Canada) supplemented with 10% fetal bovine serum (FBS) (Gibco, Canada) and 1% penicillin–streptomycin. Antibodies for flow cytometry (CD3-FITC, CD8-APC, CD56-PE, CD80-PE, CD83-APC, CD86-FITC) were from BD Biosciences. Cytokines (TNF-α, IFN-γ, IL-2, GM-CSF, IL-4) and CD3 monoclonal antibody were purchased from Peprotech (Rocky Hill, NJ, USA). Propidium iodide (PI) and 5-carboxy-fluorescein diacetate succinimidyl ester (CFSE) were purchased from Beyotime Biotechnology (Shanghai, China).

### 2.2. Generation of Dendritic Cells and CIK Cells

Fresh peripheral blood samples were collected from the umbilical cord blood according to our protocol, accepted by the local ethics committee (LEC). Peripheral blood mononuclear cells (PBMCs) were isolated by density gradient centrifugation. PBMCs at a density of 5 × 10^6^ cells/mL were incubated in a 1640 medium for 2 h at 37 °C. The adherent PBMCs were cultured in PRMI 1640 complete medium with 500 U/mL GM-CSF and 1000 U/mL IL-4 to generate DCs. The non-adherent PBMC were prepared and grown in complete RPMI 1640 medium with 1000 IU/mL rhIFN-γ (R&D Systems, Minneapolis, MN, USA). After a 24 h incubation, 50 ng/mL mouse anti-human CD3 monoclonal antibody and 1000 U/mL IL-2 were added. The CIK were incubated at 37 °C in a humidified atmosphere of 5% CO_2_ and sub-cultured every 3 days with cytokine replenishment.

### 2.3. Induction and Cultivation of Ag-DC-CIK Cells

At day 5 of DC culture, WT1, MUT1, and poly(I:C) were added to construct antigen-loaded DCs. On the 6 day, a further 500 U/mL of tumor necrosis factor α (TNF-α) was added to the DCs to induce maturation. On the 7th or 8th day of culture, WT1-MUT1-poly(I:C)-DC (Ag-DC) cells were harvested and identified. Then, matured DCs and antigen-loaded DCs were co-cultured with CIK cells in a ratio of 1:10 for an additional 7 days to harvest Ag-DC-CIK cells. Cell numbers were counted from day 3 to 7 in culture using an automated cell counter (Thermo Fisher Scientific, Waltham, MA, USA).

### 2.4. Flow Cytometry

DCs were phenotyped with a panel of antibodies: CD80-PE, CD86-FITC and HLA-DR. CIK cells were phenotyped with antibodies against CD3-FITC, CD4-FITC, CD8-APC and CD56-PE. DC-CIK cells were phenotyped with antibodies against CD3^+^CD8^+^ and CD3^+^CD56^+^. A mouse IgG (PE/FITC/APC) (BD) was used as a negative control in all the assays. Briefly, 1 × 10^6^ cells were incubated with the corresponding antibodies at 4 °C for 15 min and then washed with PBS. A total of 10,000 cells were measured and analyzed using Cell Quest Pro version 6.0 software (BD Biosciences). Dual-color flow cytometric analysis was performed using the FACSC alibur platform (BD Biosciences, San Jose, CA, USA).

### 2.5. LDH Cytotoxic Assay

Killing of target cells was measured in lactate dehydrogenase (LDH)-release assay (Promega, Madison, WI, USA). LDH-release was measured in an enzymatic assay according to the manufacturers’ protocol. Death of target cells or % cytotoxicity was calculated was follows (% Cytotoxicity = (Experimental − Effector Spontaneous − Target Spontaneous × 100)/(Target Maximum − Target Spontaneous), in which all values were normalized by subtraction of the LDH backgrounds originating from the spontaneous release of effector cells.

### 2.6. Cell Apoptosis Assay

For apoptosis analysis, annexin V/propidium iodide (PI) double staining was performed according to the instruction manual of the Annexin V-FITC Apoptosis Detection Kit (Beyotime Biotechnology, Shanghai, China). Briefly, adherent cells were digested using trypsin. The cells were washed with phosphate-buffered saline (PBS) once, centrifuged to remove residual body liquid and gently resuspended in 195 μL of Annexin V-FITC binding solution. Next, 5 μL of Annexin V/FITC was added, mixed gently, followed by 5 μL of propidium iodide (PI), and incubated at room temperature in the dark for 20 min. The cells were then detected using a flow cytometer (Beckman, Brea, CA, USA).

### 2.7. Cell Migration Assay

Tumor cell migration was measured using the transwell cell migration assay. In brief, cells suspended in 200 μL serum-free DMEM or PRIM 1640 media were seeded into the upper chamber of the transwell. The number of cells was counted using a light microscope in five random fields (Olympus Corporation, Tokyo, Japan).

### 2.8. Establishment of Tumor-Derived Gastric Organoids

Gastric organoids were established as previously described [17]. Tumor tissues were washed 20–30 times with ice-cold PBS until they were free of impurities. Biopsies were then dissociated to single cells using a Human Tumor Dissociation Kit (Miltenyi Biotec, Bergisch Gladbach, Germany) for 1 h at 37 °C. Dissociated cells were filtered through a 70-μm cell strainer (BD Biosciences, San Jose, CA, USA) into a new 50 mL tube. To remove red blood cells, the filtered cells were incubated with ACK lysis buffer for 5 min and washed with basal media (DMEM/F12, 10 mM HEPES, 1 × Gluta MAX, 1 × penicillin/streptomycin). The cells were resuspended in 30 μL of growth factor-reduced Matrigel (Corning, NY, USA) and seeded in 4-well dishes. Matrigel was then solidified by a 15 min incubation at 37 °C and overlaid with 500 μL of complete organoid media. Complete media were refreshed at 2–3 day intervals; passaging of gastric organoids was performed using a cell recovery solution (Corning, Inc., Corning, NY, USA) once every two weeks with a split ratio of 1:4.

### 2.9. Tumor Formation Assay in Nude Mice

All animal experiments were approved by the Animal Experimental Ethics Committee of Henan Agricultural University Institutional Animal Care and Use Committee (approval No. HNND20191201). MKN45 cells were grown to confluence in 250 mL flasks. Next, cells were suspended in sterile saline to a concentration of 1 × 10^7^/mL. To establish the xenograft tumor model, cell suspension (100 μL/mouse) was subcutaneously injected into the left flank of 8-week-old male nude mice. After tumor growth on day 14, the mice were randomly divided into 4 groups with similar tumor sizes. The first group served as the control group without any treatment (MKN45-control, n = 6). The other 3 groups were subjected to tail vein injections of CIK (2 × 10^6^ cells, n = 6), DC-CIK (2 × 10^6^ cells, n = 6) and MT1/MUC1/poly(I:C)-loaded DC-CIK (2 × 10^6^ cells, n = 6) dissolved in 100 μL of 0.9% saline, respectively. Mice were administered 3 times a week for 3 weeks. In addition to the tumor weights, the subcutaneous tumor volume was measured daily later on day 21 after tumor initiation, using a caliper and volume was estimated as follows: Tumor volume (mm^3^) = 0.5 × length × width^2^. Mice were sacrificed on day 21, tumors were dissected, weighed, and snap-frozen for Western blot and immunohistochemistry analysis.

### 2.10. Statistical Analysis

Values are expressed as the mean ± standard deviations of the mean. Statistical analysis was performed using Student’s *t*-test or one-way analysis of variance. Statistical analyses were performed using SPSS 16.0 software. *p* ≤ 0.05 was considered to indicate a statistically significant difference.

## 3. Results

### 3.1. Induction and Identification of DC and CIK

**Figure 1:**
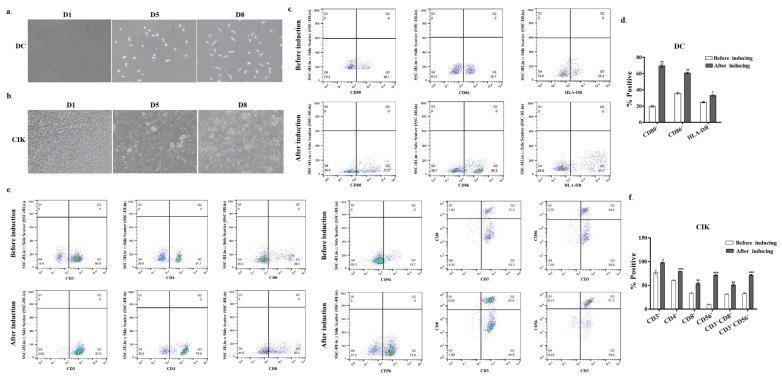
Morphological characterization and phenotypical analysis of DC and CIK maturation in vitro. (**a**) PBMCs on day 1 and DCs cultured in the presence of GM-CSF, IL-4, or TNF-α for 8 days; (**b**) CIK cells from non-adherent PBMCs cultured in the presence of IFN-γ and IL-2. Cells were imaged using DIC microscopy (Carl Zeiss, Munich, Germany) for visualization; (**c**) DCs cultured in a cytokine-enriched medium were incubated with CD80-PE, CD86-FITC, and HLA-DR antibodies, and the phenotypes of these cells were analyzed by FCM; (**d**) the quantification of CD80^+^, CD86^+^ and HLA-DR expression for 8 days in culture is given as mean ± SE from three independent experiments (right). * *p* < 0.05, ** *p* < 0.01; (**e**) CIK cells cultured for 8 days were incubated with CD3-FITC, CD4-FITC, CD8-APC, and CD56-PE antibodies. The image showed the FCM data for the CD3^+^, CD4^+^, CD8^+^, CD3^+^CD8^+^, CD3^+^CD56^+^ expression after 8 days in culture; (**f**) the quantification of CD3^+^, CD4^+^, CD8^+^, CD3^+^CD8^+^, CD3^+^CD56^+^ expression for 7 and 15 days in culture is given as mean ± SE from three independent experiments. * *p* < 0.05; ** *p* < 0.01; *** *p* < 0.001.

Mature and active DCs were generated in vitro from peripheral blood mononuclear cells (PBMCs) obtained from human umbilical cord blood. The differentiation process of these cells was monitored by evaluating morphological changes over time. The mature phenotype of DCs was confirmed via flow cytometry (FCM) analysis, focusing on cell surface markers such as CD80, CD86, and HLA-DR. Initially, freshly isolated PBMCs appeared spherical and small, exhibiting smooth surfaces without protrusions, as observed under light microscopy (Figure 1a). Following 3 days of culture in cytokine-enriched media, the adherent cells increased in size and adopted an oval morphology. By day 5, the cells detached from the plastic substrate and transitioned to suspension growth, displaying circular or irregular shapes with small dendritic pseudopodia extending from the cell membrane (Figure 1a). Prolonged culture further enhanced the development of dendritic protrusions, ultimately resulting in the characteristic dendritic morphology of mature DCs (Figure 1a DC middle and right images). This morphological transformation was associated with an increased expression of cell surface markers. By day 8 of culture, FCM analysis indicated a significant upregulation of surface markers compared to day 1. Specifically, the proportion of CD80^+^ cells increased from 20.7% to 71.2% (*p* < 0.001), CD86^+^ from 36.7% to 60.3% (*p* < 0.001), and HLA-DR from 25.4% to 34.1% (*p* < 0.05) (Figure 1c,d). These findings suggest that DCs achieved a mature phenotype following 8 days of culture.

To generate CIK cells in vitro, the non-adherent PBMCs were isolated and cultured in the presence of interferon-γ (IFN-γ), CD3 monoclonal antibody, IL-2, and other cytokines. As shown in Figure 1b, cells exhibited a five-fold increase in average cell area on day 5 of culture, compared to that on day 1, displaying irregular morphology. Multiple cells formed clusters and aggregated into cell conglomerates (Figure 1b, middle and right images). After 8 days of culture, cell morphology features confirmed CIK cell maturation (Figure 1b). Subsequently, flow cytometry analysis revealed significant increases in key effector subsets by day 8 versus day 1 (Figure 1e). Specifically, the proportions of cells expressing CD3^+^, CD4^+^, CD8^+^, CD56^+^, CD3^+^CD8^+^, and CD3^+^CD56^+^ were all significantly increased in cytokine-enriched culture media on day 8 compared to that on day 1 (Figure 1e). Particularly, the percentage of CD3^+^CD8^+^ cells (from 31.4% to 52.6%) and CD3^+^CD56^+^ cells (from 34.4% to 71.3%) showed marked enrichment. These findings were consistent with the morphological maturation profile in Figure 1b, further confirming the successful generation of mature CIK cells.

### 3.2. Effects of DC-CIK on Lymphocyte Subsets and Proliferation

**Figure 2:**
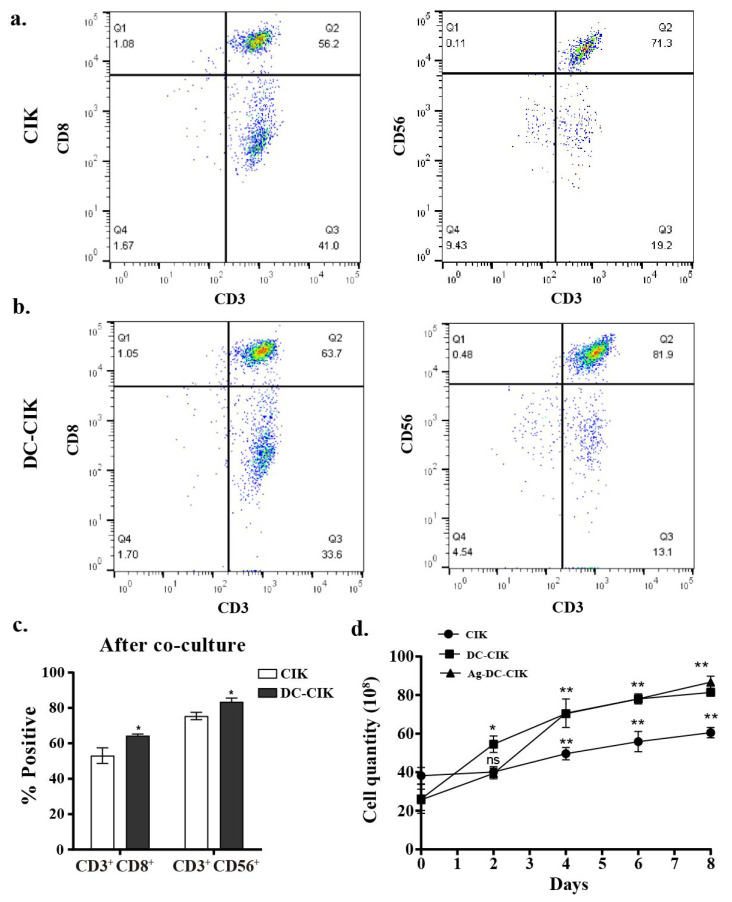
Enhanced cytotoxic cell populations after DC-CIK co-culture. (**a**,**b**) Flow cytometry analysis of CIK cells cytotoxic subsets (CD3^+^CD8^+^ and CD3^+^CD56^+^) before and after CIK cell co-culture; (**c**) statistical quantification of cytotoxic population expansion; (**d**) proliferation kinetics of CIK, DC-CIK, and Ag-DC-CIK cells. DC, dendritic cell; CIK, cytokine-induced killer; Ag-DC-CIK, poly(I:C)-MUC1-loaded DC-CIK. Data represent mean ± SEM of three independent experiments. ns, no significance; * *p* < 0.05; ** *p* < 0.01.

Following the co-culture of DC with CIK cells, the proportions of lymphocyte subsets on the surface, including CD8^+^ T cells (CD3^+^CD8^+^) and NKT-like cells (CD3^+^CD56^+^), were analyzed by flow cytometry. The results indicated an increase in the percentages of both CD8^+^ T and NKT-like cells. Specifically, over the culture period from day 0 to day 8, the proportion of CD3^+^CD8^+^ cells increased by 21.1% (from 52.6% to 63.7%), while that of CD3^+^ CD56^+^ cells increased by 14.9% (from 71.3% to 81.9%) (Figure 2a–c). These elevated proportions of lymphocyte subset versus CIK-only controls indicate enhanced lymphocyte activation in DC-CIK systems.

Furthermore, cell counting revealed that DC-CIK and Ag-DC-CIK cells (WT1/MUC1-loaded, 1:1 ratio) exhibited significantly higher proliferation rates compared to CIK cells alone (*p* < 0.001) (Figure 2d). Although the Ag-DC-CIK group showed a tendency toward enhanced proliferation relative to unloaded DC-CIK, the difference between these two groups was not statistically significant. These results indicate that the presence of DCs, whether antigen-loaded or not, substantially promotes CIK expansion in vitro. The similar proliferation capacity between DC-CIK and Ag-loaded DC-CIK further supports the potential of DC-based CIK immunotherapy in targeting cancer cells, although the underlying mechanisms remain complex.

### 3.3. Antitumor Activity of Ag-DC-CIK Cells In Vitro

**Figure 3:**
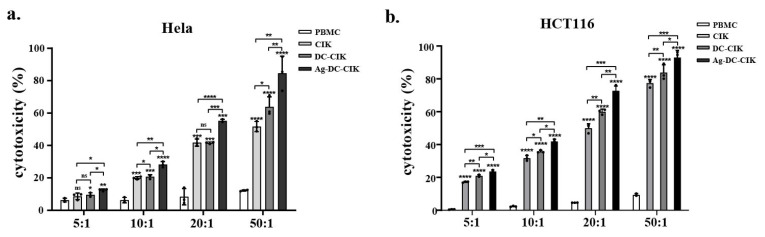

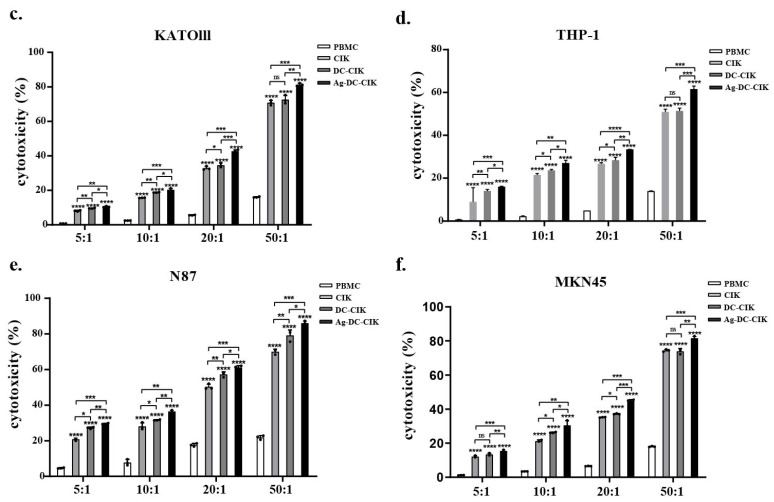
Cytotoxic effects of effector cells against multiple cancer cell lines assessed by LDH release assay. (**a**–**f**) Dose-dependent killing activities of CIK, DC-CIK and Ag-DC-CIK cells against cervical (HeLa), colorectal (HCT116), gastric (MKN45/KATOIII/N87) and acute leukemia (THP-1) cell lines at varying effector-to-target ratios (5:1, 10:1, 20:1 and 50:1). Uninduced PBMCs served as negative control. Data represent mean ± SEM of three independent experiments. ns, no significance; * *p* < 0.05; ** *p* < 0.01; *** *p* < 0.001; **** *p* < 0.0001.

The cytotoxic effects of CIK, DC-CIK, and Ag-DC-CIK cells against various tumor cell lines were assessed using a lactate dehydrogenase (LDH) release assay. Uninduced PBMCs served as the control, while CIK, DC-CIK, and Ag-DC-CIK cells functioned as effector cells. The target cells comprised several tumor cell lines, including HeLa (cervical cancer), HCT116 (colorectal cancer), MKN45/KATOIII/N87 (gastric cancer), and THP-1 (acute monocytic leukemia). At an effector-to-target (E:T) ratio of 5:1, CIK cells exhibit minimal cytotoxicity against HeLa cells compared to the control. In contrast, both DC-CIK and Ag-DC-CIK cells demonstrated significantly enhanced cytotoxic activity (*p* < 0.05). The cytotoxic effects of all effector cells escalated with increasing E: T ratios (10:1, 20:1, 50:1), with Ag-DC-CIK cells consistently exhibiting superior killing efficacy against all the tumor types, relative to both CIK and DC-CIK cells (*p* < 0.05; Figure 3a–f). Notably, Ag-DC-CIK achieved the highest tumoricidal activity at all ratios, demonstrating statistically superior efficacy versus other effectors (*p* < 0.05 for all comparisons). These findings indicate that Ag-DC-CIK therapy exhibits a broad-spectrum antitumor potential and demonstrates enhanced cytotoxicity compared to traditional CIK and DC-CIK approaches.

### 3.4. Pro-Apoptotic Effects of Ag-DC-CIK on Various Tumor Cells

**Figure 4:**
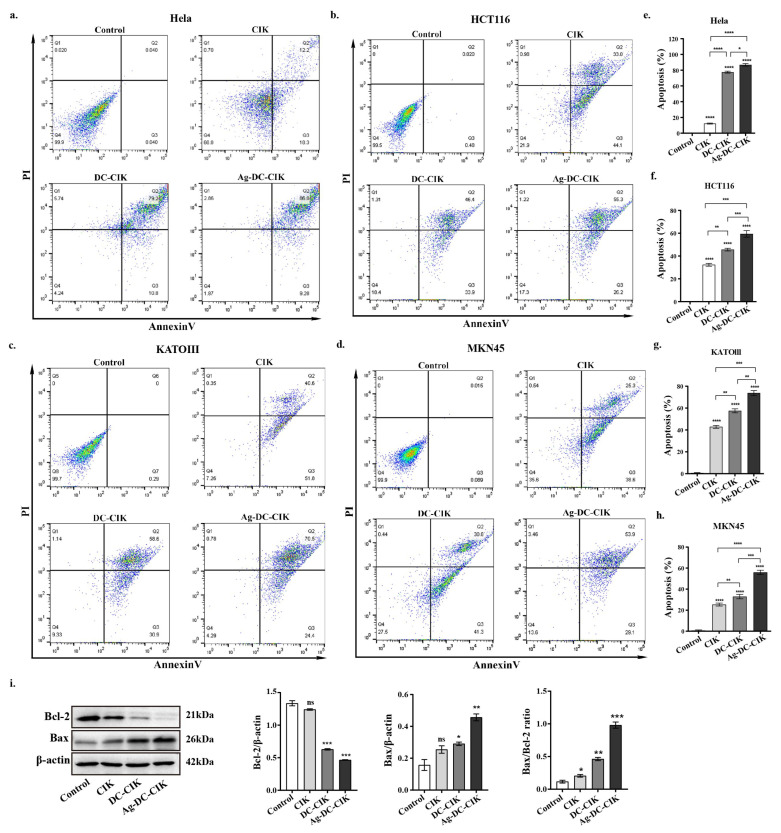
Comparative analysis of apoptosis induction by effector cells against multiple cancer cell lines evaluated by Annexin V-FITC/PI staining. (**a**–**d**) Representative flow cytometry dot plots showing Annexin V-FITC/PI staining of (**a**) cervical (HeLa), (**b**) colorectal (HCT116), (**c**) gastric (MKN45/KATOIII), and (**d**) acute leukemia (THP-1) cancer cells after 24 h co-culture with CIK, DC-CIK, or Ag-DC-CIK cells (effector-to-target ratio 20:1). (**e**–**h**) Quantitative analysis of apoptotic cell populations. (**i**) Western blot analysis of apoptosis-related proteins Bcl-2 and Bax, with densitometric quantification normalized to β-actin (*p* < 0.05 vs. other groups). ns, no significance; * *p* < 0.05; ** *p* < 0.01; *** *p* < 0.001; **** *p* < 0.0001.

To assess the pro-apoptotic effects of CIK, DC-CIK, and Ag-DC-CIK cells on tumor cells, the apoptosis level of the targets was evaluated using Annexin V-FITC/PI staining and flow cytometry after 24 h of co-culture with the effector cells. In co-culture experiments involving HeLa cells, all effector cell groups (CIK, DC-CIK, and Ag-DC-CIK) induced significantly higher apoptosis levels than the control group (*p* < 0.0001) (Figure 4a,e). Among these, Ag-DC-CIK cells exhibited the most pronounced pro-apoptotic effect, showing statistically significant differences compared to both CIK and DC-CIK cells. Similar results were observed in HCT116 cells, where Ag-DC-CIK cells demonstrated the greatest apoptosis-inducing capacity (*p* < 0.001 vs. CIK; *p* < 0.001 vs. DC-CIK) (Figure 4b,f). In gastric cancer cell lines (KATO III and MKN45), all effector cell types significantly enhanced apoptosis relative to the control, with Ag-DC-CIK cells again displaying superior efficacy (Figure 4c,d,g,h).

Additionally, Western blot analysis was performed to assess the expression levels of apoptosis-related proteins, Bax and Bcl-2. The results supported these observations, showing that Ag-DC-CIK treatment significantly upregulated the pro-apoptotic protein Bax (*p* < 0.01) and notably downregulated the anti-apoptotic protein Bcl-2 (*p* < 0.05) in MKN45 cells (Figure 4i). This result was confirmed in HCT116 cells (Figure S2). Taken together, these findings demonstrate the enhanced pro-apoptotic efficacy of Ag-DC-CIK cells across multiple cancer cell lines.

### 3.5. Ag-DC-CIK Inhibits Cancer Cell Migration

**Figure 5:**
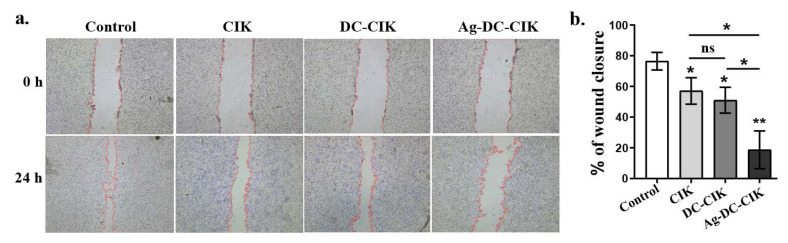
Inhibitory effects of effector cells on gastric cancer cell migration assessed by a wound healing assay. (**a**) Representative images of wound closure in MKN45 gastric cancer cells cultured alone (Control) or co-cultured with CIK, DC-CIK, or Ag-DC-CIK cells at 0 h and 24 h post-scratching. Dashed lines indicate initial wound boundaries. (**b**) Quantitative analysis of migration rates. ns, no significance; * *p* < 0.05; ** *p* < 0.01.

Cell migration is a hallmark of cancer invasion and metastasis [18,19]. To examine the effects of Ag-DC-CIK on gastric cancer cell migration, we performed a wound healing assay using MKN45 cells to measure the percentage of wound area closure. The results demonstrated that Ag-DC-CIK significantly suppressed the migratory capacity of MKN45 cells compared to the CIK and DC-CIK groups, after 24 h of co-culture (Figure 5a). Quantitative analysis further confirmed this inhibitory effect, showing that the rates of wound closure in the CIK, DC-CIK, and Ag-DC-CIK groups were all significantly reduced (*p* < 0.05) compared to the control group, with the Ag-DC-CIK group exhibiting the lowest wound closure rate among all groups (Figure 5b). These results were also reproducible in HCT116 cells (Figure S3). These findings indicate that Ag-DC-CIK markedly inhibits the migration of gastric cancer cells, suggesting its potential to suppress the progression of gastric cancer.

### 3.6. Inhibitory Effects of Ag-DC-CIK Cells on Gastric Cancer Organoids

**Figure 6:**
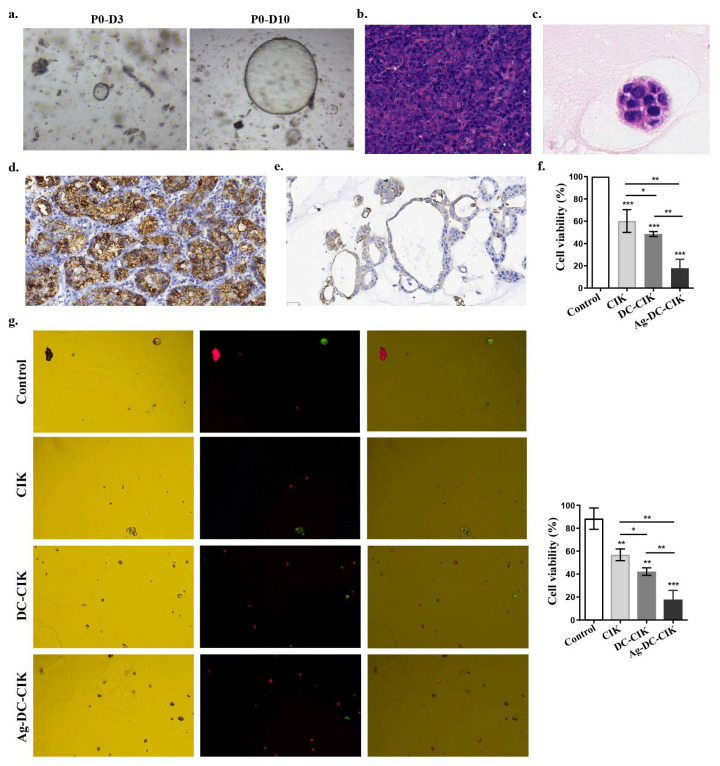
Therapeutic effects of Ag-DC-CIK on gastric cancer organoids. (**a**) Morphological development of patient-derived gastric cancer organoids during 10-day culture (×200). (**b**,**c**) H&E staining of gastric cancer tissue (×200) (**b**) and organoids (×400) (**c**). (**d**,**e**) Immunohistochemical analysis of CDX2 expression in primary gastric cancer tissue (scar bar: 30 μm) (**d**) and derived organoids (scar bar: 30 μm) (**e**). (**f**) The cytotoxic effects of effector cells against gastric cancer organoids by CCK-8 viability assay. (**g**) The apoptotic cell death was analyzed in organoids treated with Ag-DC-CIK versus other groups by AO/PI dual staining (×200). Data represent mean ± SEM of three independent experiments. * *p* < 0.05; ** *p* < 0.01; *** *p* < 0.001.

Initially, gastric cancer (GC) appears as clumped cells in the three-dimensional matrix. Within 3–4 days, these cells develop into spherical cystic bodies, with a smooth surface and glossy appearance (Figure 6a). The organoids continued to expand, displaying vacuolated structures by day 7, and reached diameters exceeding 300 μm by day 10, typically being arranged in clusters with irregular clumps or structures (Figure 6a). Histological analysis using H&E staining revealed that gastric cancer tissues featured inflammatory cell infiltration and glandular-like structures, characterized by enlarged, hyperchromatic nuclei and an increased nuclear-to-cytoplasmic ratio (Figure 6b). In contrast, gastric cancer organoids comprised a monolayer of epithelial cells with comparable nuclear atypia (Figure 6c).

Immunohistochemical staining confirmed high CDX2 expression, a known prognostic factor in gastric cancer, in both primary gastric cancer tissues (Figure 6d) and organoids (Figure 6e), consistent with its established role in intestinal metaplasia and gastric adenocarcinoma progression. Additionally, the results of the CCK-8 assay demonstrated that CIK, DC-CIK, and Ag-DC-CIK cells exhibited significant cytotoxicity against GC organoids (*p* < 0.0001). Notably, the Ag-DC-CIK cells exhibited a superior killing efficacy compared to both CIK and DC-CIK cells (*p* < 0.01) (Figure 6f). Further validation via AO-PI dual staining confirmed that Ag-DC-CIK treatment resulted in the lowest cell viability in GC organoids, significantly outperforming both CIK and DC-CIK cells (*p* < 0.01) (Figure 6g).

### 3.7. In Vivo Antitumor Efficacy and Safety Evaluation of Ag-DC-CIK in Gastric Cancer Xenograft Models

**Figure 7:**
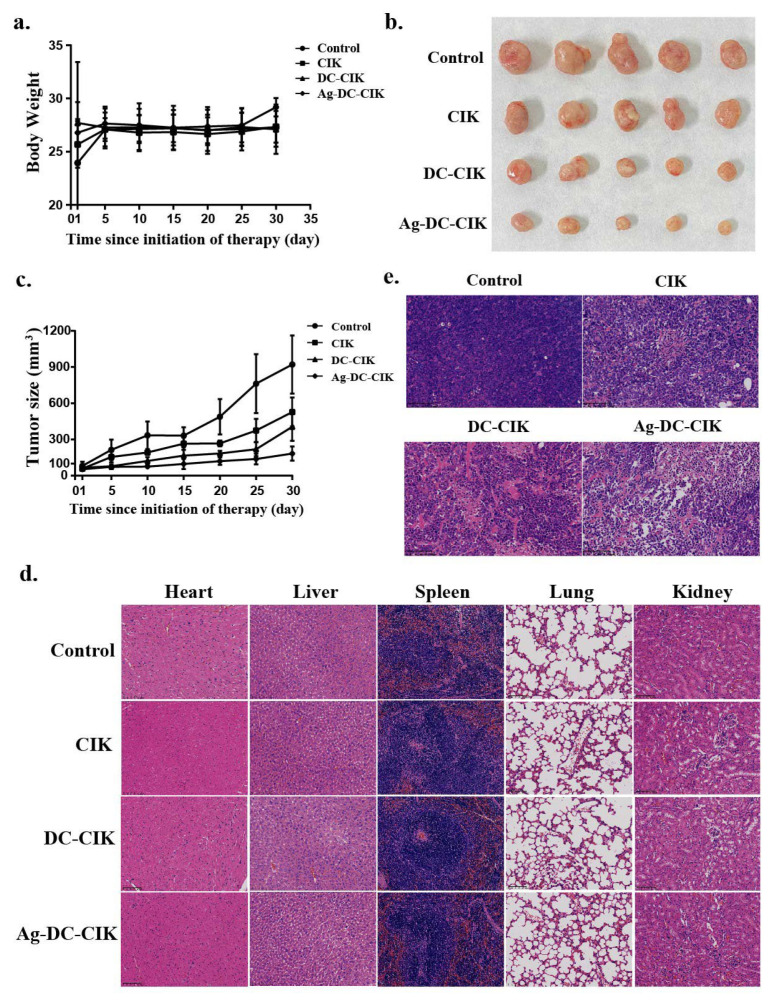

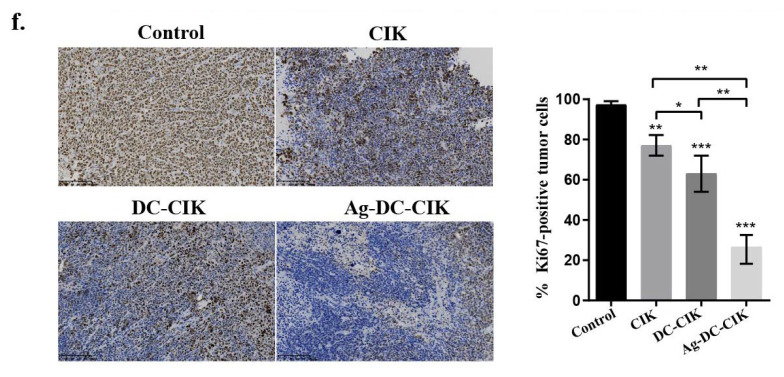
In vivo antitumor efficacy and safety evaluation of Ag-DC-CIK in xenograft nude mouse models. (**a**) Body weight of xenograft mouse was observed in CIK, DC-CIK, and Ag-DC-CIK treatment groups. (**b**) Representative images of excised tumors from control and treatment groups. (**c**) Tumor growth curves of Ag-DC-CIK treatment compared to CIK, DC-CIK, and PBS control (n = 6 per group). (**d**) H&E staining of major organs (heart, liver, spleen, lung, kidney) were analyzed in all groups (scale bar: 100 μm). (**e**) Tumor histopathology of Ag-DC-CIK group (scar bar: 100 μm). (**f**) Immunohistochemistry staining of Ki67 in tumors from control and Ag-DC-CIK treatment groups (scar bar: 100 μm). Data represent mean ± SEM of three independent experiments. * *p* < 0.05, ** *p* < 0.01, *** *p* < 0.001.

To further evaluate the therapeutic efficacy of Ag-DC-CIK cells on cancers, nude mice were subcutaneously injected with 2 × 10^6^ MKN45 gastric cancer cells into the left axilla. After 10 days of tumor formation, mice with tumor volumes reaching 50 mm^3^ were administered CIK, DC-CIK, or Ag-DC-CIK cells via tail vein injection three times weekly for three weeks. Observational data revealed that from days 25 to 30, the Ag-DC-CIK group exhibited significantly greater body weight gain compared to the CIK and DC-CIK groups (Figure 7a). Tumor size assessments were measured regularly until the endpoint at day 30 post-treatment initiation, it was found that Ag-DC-CIK treatment induced a marked reduction in tumor volume relative to the CIK and DC-CIK groups (Figure 7b). Correspondingly, the tumor growth curves further demonstrated consistently smaller tumor volumes in the Ag-DC-CIK group at all timepoints (*p* < 0.05 vs. control group; Figure 7c). These findings suggest that Ag-DC-CIK therapy effectively mitigates gastric cancer progression in vivo.

To assess the safety and antitumor efficacy of Ag-DC-CIK cells, histopathological analysis was performed using hematoxylin and eosin (H&E) and immunohistochemistry (IHC) staining. The results of H&E analysis demonstrated that no significant pathological alterations were observed in major organs in the nude mice, including heart, liver, spleen, lung, and kidney (Figure 7d). Meanwhile, in the Ag-DC-CIK treatment group, a reduction in tumor malignancy was observed, as characterized by decreased tumor cell density, a reduced tumor cell count, and the presence of extensive areas of tumor necrosis (Figure 7e).

Ki67 is a well-established cell proliferation marker associated with tumor progression. The IHC analysis of Ki67 revealed significantly reduced Ki67-positive cell rates in the CIK, DC-CIK, and Ag-DC-CIK groups, as compared to the control. Critically, the Ag-DC-CIK group showed markedly greater suppression of Ki67 expression versus the CIK and DC-CIK groups (*p* < 0.001; Figure 7f). Collectively, these findings establish the enhanced therapeutic potential of Ag-DC-CIK cells against gastric cancer, attributed to their robust antitumor activity and favorable safety profile.

## 4. Discussion

Over the past two decades, advancements in tumor immunology and molecular biology have fundamentally transformed cancer treatment paradigms, with immunotherapy establishing itself as a pivotal approach for enhancing patient outcomes [20,21]. Among adaptive cell therapies, CIK cells constitute a distinct population that integrates the cytotoxic efficacy of T lymphocytes and natural killer (NK) cells. This integration offers several advantages, including substantial in vitro expansion, broad-spectrum antitumor activity, and minimal off-target toxicity [22,23]. The antitumor effectiveness of CIK cells is predominantly facilitated by the CD3^+^CD56^+^ NKT subset, known for its remarkable proliferative and cytolytic capabilities [2]. DCs, as principal antigen-presenting cells, enhance this response by priming native T cells and initiating adaptive immunity [1]. The ex vivo co-culture of DCs and CIK cells (DC-CIK therapy) thus emerges as a promising strategy to exploit synergistic immune activation, as their interaction promotes a coordinated and sustained antitumor response [24].

In this study, we developed a multi-antigen-loaded DC-CIK (Ag-DC-CIK) system incorporating WT1, MUC1, and poly(I:C), which substantially improved antitumor efficacy through synergistic immune modulation. Flow cytometry analysis confirmed the successful induction of DC-CIK, as evidenced by elevated expression of DC maturation markers (CD80^+^/CD86^+^/HLA-DR) and the expansion of cytotoxic CIK subsets (CD3^+^CD56^+^). We compared the CIK cells, DC-CIK cells, and Ag-DC-CIK cells. We found that Ag-DC-CIK cells significantly enhanced the antitumor activity, increased the proliferation capacity of CIK cells, and maintained the activity of CIK cells. The establishment of Ag-DC-CIK cells provided a basis for a new, safe, and effective immunotherapy. Importantly, Ag-DC-CIK demonstrated superior cytotoxicity against a variety of tumor types compared to conventional DC-CIK, likely attributable to the following three principal mechanisms. (1) Poly(I:C)-induced DC maturation: Activation of TLR3 enhanced antigen cross-presentation and Th1-polarized cytokine secretion [25,26], thereby priming CIK cells for enhanced effector function. (2) Expanded TCR repertoire: The dual-antigen loading (WT1 + MUC1) potentially diversifies T-cell recognition, addressing the challenge of tumor heterogeneity, which is a limitation of single-antigen approaches. (3) Induction of mitochondrial apoptosis: Ag-DC-CIK treatment significantly increased the Bax/Bcl-2 ratio, indicating enhanced activation of intrinsic apoptotic pathways.

To assess clinical relevance, we utilized patient-derived gastric cancer organoids, which accurately replicate the cellular heterogeneity, tumor–stroma interactions, and drug resistance mechanisms characteristic of primary tumors [27,28]. The application of Ag-DC-CIK therapy significantly diminished organoid viability in comparison to unmodified DC-CIK, corroborating previous findings related to antigen-specific T cells in colorectal cancer organoids [29]. Notably, our study is the first to incorporate poly(I:C)-primed DCs, which may enhance therapeutic efficacy through the recruitment of immune cells driven by IFN-γ. These findings highlight the potential of organoids as predictive platforms for the optimization of immunotherapy.

In vivo studies have shown that Ag-DC-CIK therapy exhibits significant tumor suppression in gastric cancer xenografts, as evidenced by histopathological analysis indicating extensive necrosis and a reduction in Ki67+ proliferative cells. This therapeutic approach surpasses previous findings related to DC-CIK monotherapy, likely attributable to poly(I:C)-mediated activation of NK cells and antigen spreading. The lack of systemic toxicity, as indicated by preserved organ histology, further underscores the potential clinical applicability of this strategy. Nonetheless, the use of immunocompromised nude mice limits the assessment of adaptive immune contributions, highlighting the need for future research utilizing humanized mouse models to comprehensively evaluate T-cell responses.

## 5. Conclusions

This study presents a novel MT1/MUC1/poly(I:C)-loaded DC-CIK system that addresses critical limitations of traditional DC-CIK therapy and improves antitumor effectiveness. The results obtained from patient-derived organoids and xenograft models highlight the potential of this approach for clinical application, especially in the context of heterogeneous malignancies such as gastric cancer. This research not only progresses the field of DC-CIK therapy, but also establishes a foundation for combinatorial antigen engineering in cancer immunotherapy.

## Data Availability

All data generated or analyzed during this study are included in this article.

## Supplementary Materials

The following supporting information can be downloaded at https://www.mdpi.com/article/10.3390/biom15101356/s1, Figure S1: Uncropped Western blots for Figure 4i; Figure S2: Western blot analysis of apoptosis-related proteins Bcl-2 and Bax in HCT116 cells, with densitometric quantification normalized to β-actin levels; Figure S3: (a) Representative images of wound closure in HCT116 cells cultured alone (Control) or co-cultured with CIK, DC-CIK, or Ag-DC-CIK cells at 0 h and 24 h post-scratching. Dashed lines indicate initial wound boundaries. (b) Quantitative analysis of migration rates; Figure S4: Uncropped Western blots for Figure S2.

## Abbreviations

The following abbreviations are used in this manuscript:

WT1	Wilms’ tumor 1 gene
MUC1	Mucin 1
CCK-8	Cell counting kit-8
CIK	Cytokine-induced killer
DC	Dendritic cells
MHC	Major histocompatibility complex
NK	Natural killer
PBMCs	Peripheral blood mononuclear cells
TNF-α	Tumor necrosis factor α
rhIFN-γ	Recombinant human interferon gamma
