# Supplementary figures and images for: Dendritic Cell-Cytokine-Induced Killer Cells Co-Loaded with WT1/MUC1/Poly(I:C) Enhance Antitumor Immune Responses In Vitro and In Vivo
Source: Biomolecules. 2025 Sep 24;15(10):1356. doi: 10.3390/biom15101356 (PMC12564647; doi:10.3390/biom15101356)

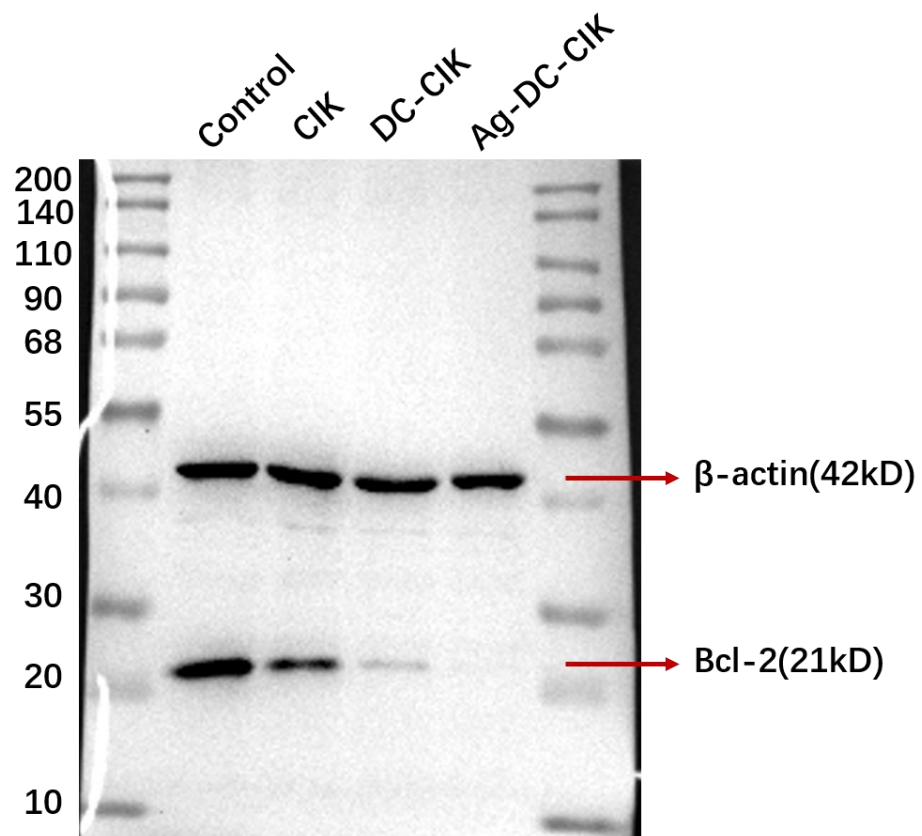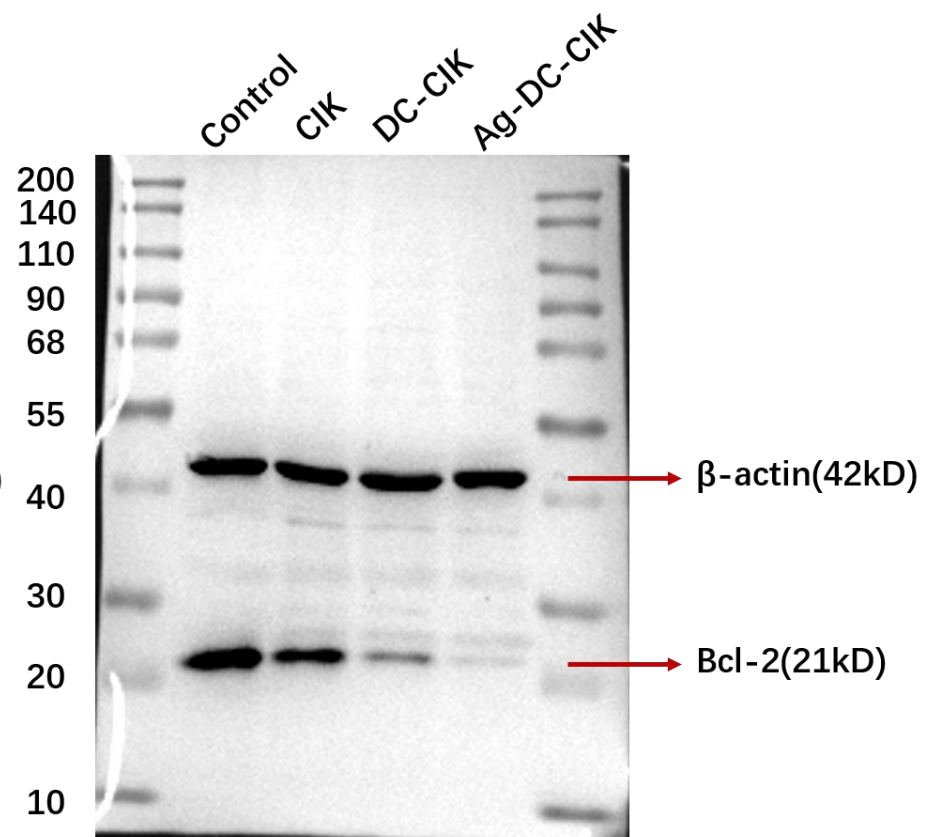

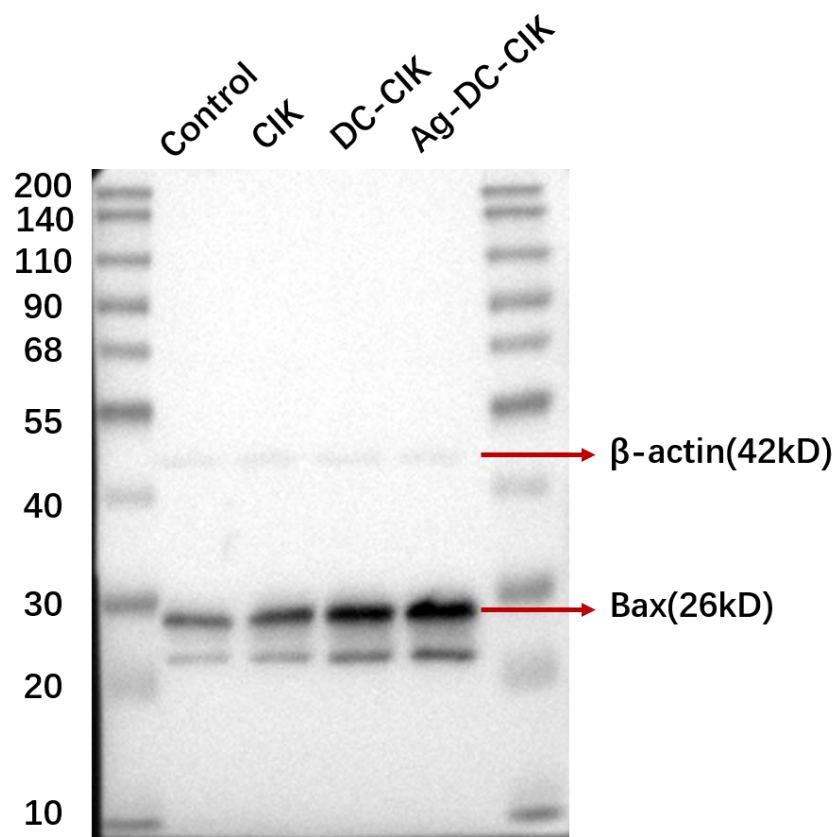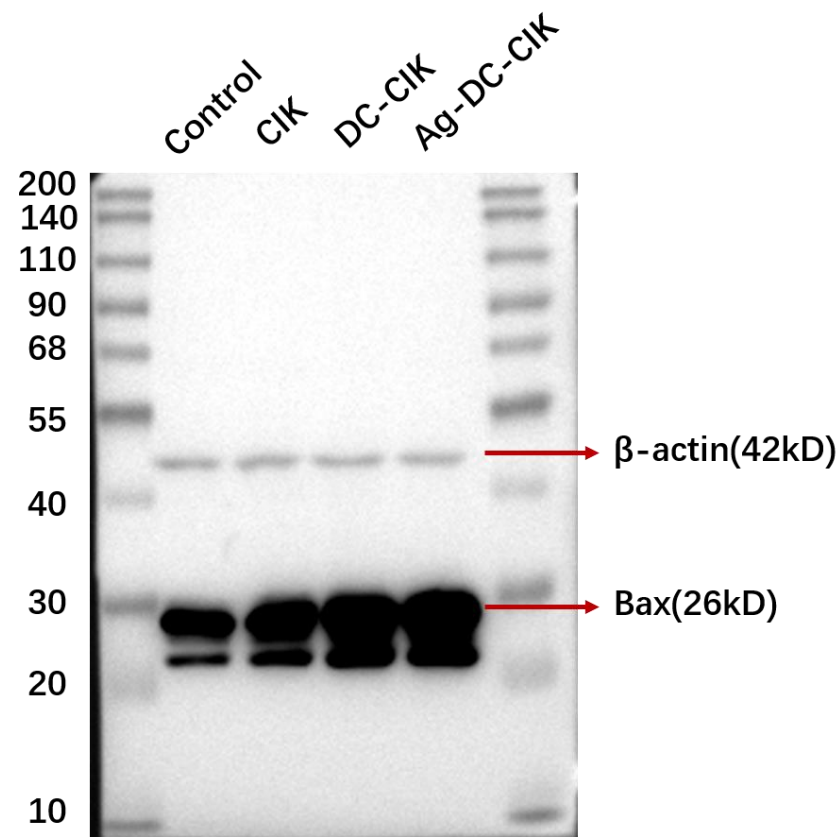

Uncropped western blots for figure 4i are found in Supplementary Figures S1.

Supplement: Supplementary file 1 [file biomolecules-15-01356-s001.zip › Figure S1.pdf]

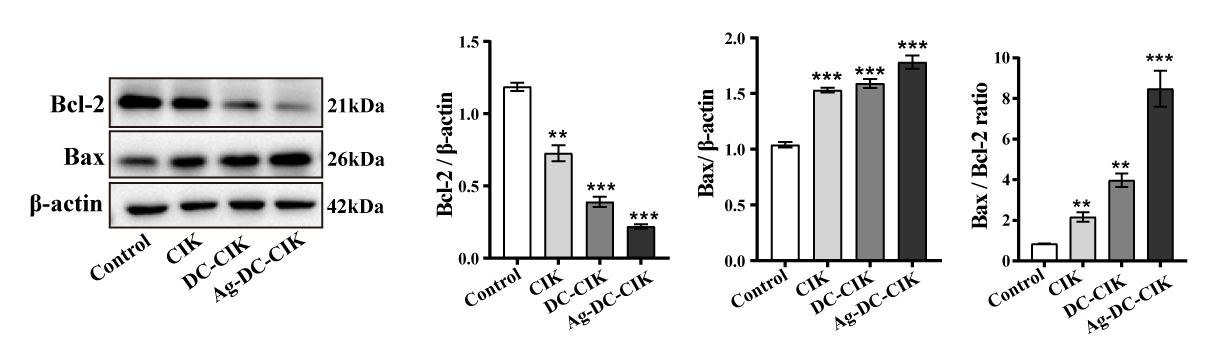

Supplement: Supplementary file 1 [file biomolecules-15-01356-s001.zip › Figure S2.tif]

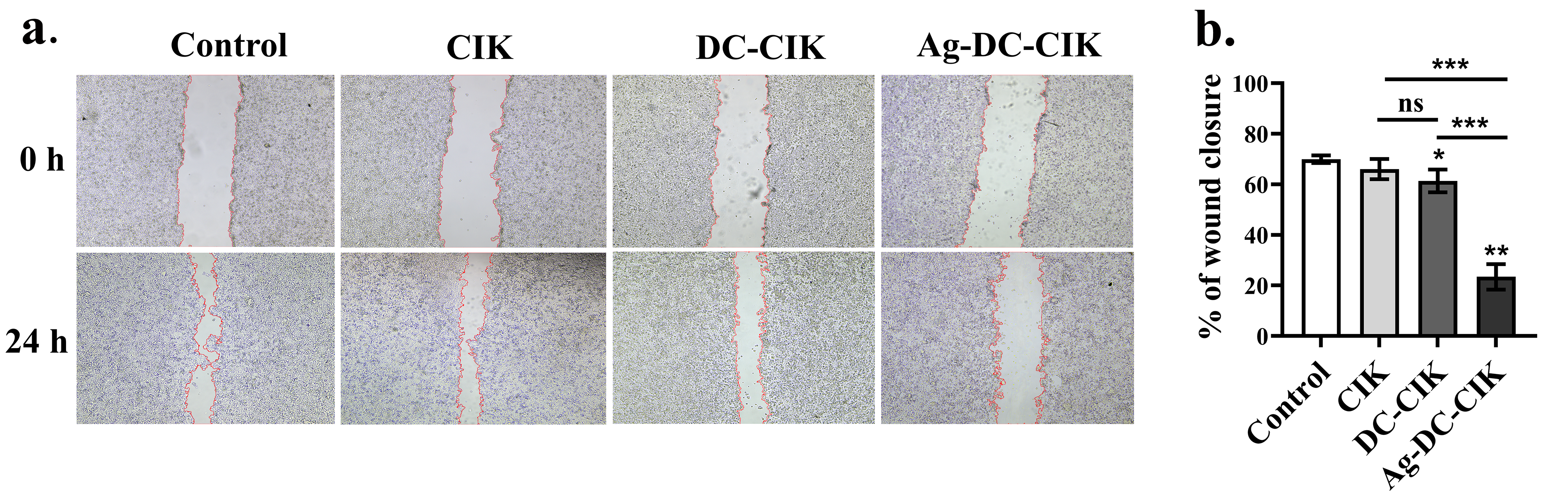

Supplement: Supplementary file 1 [file biomolecules-15-01356-s001.zip › Figure S3.tif]

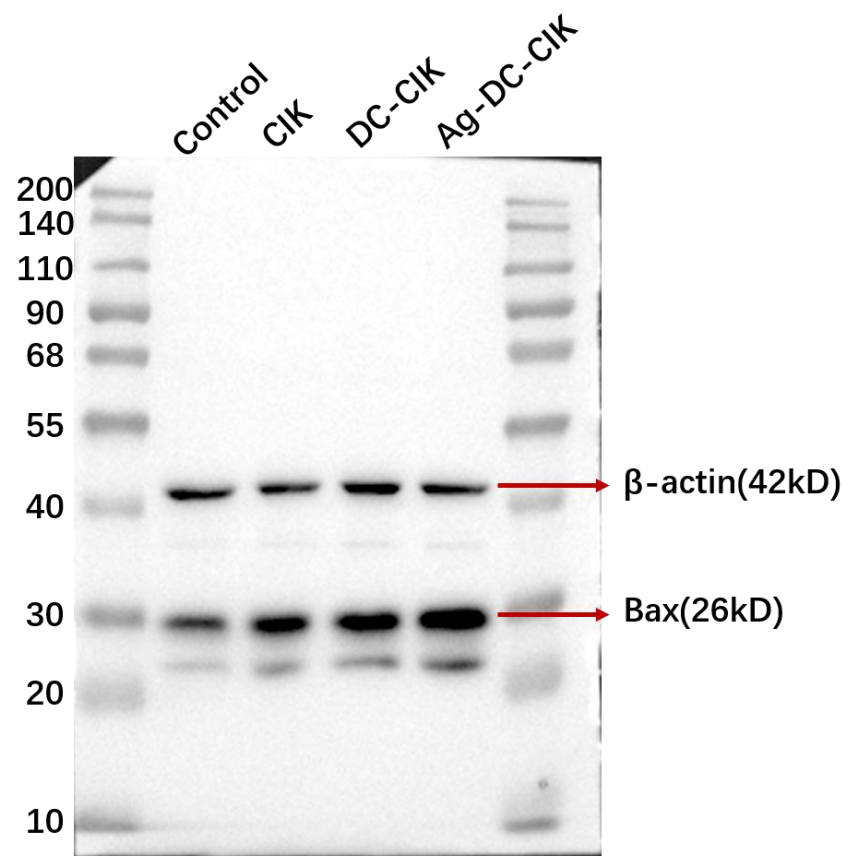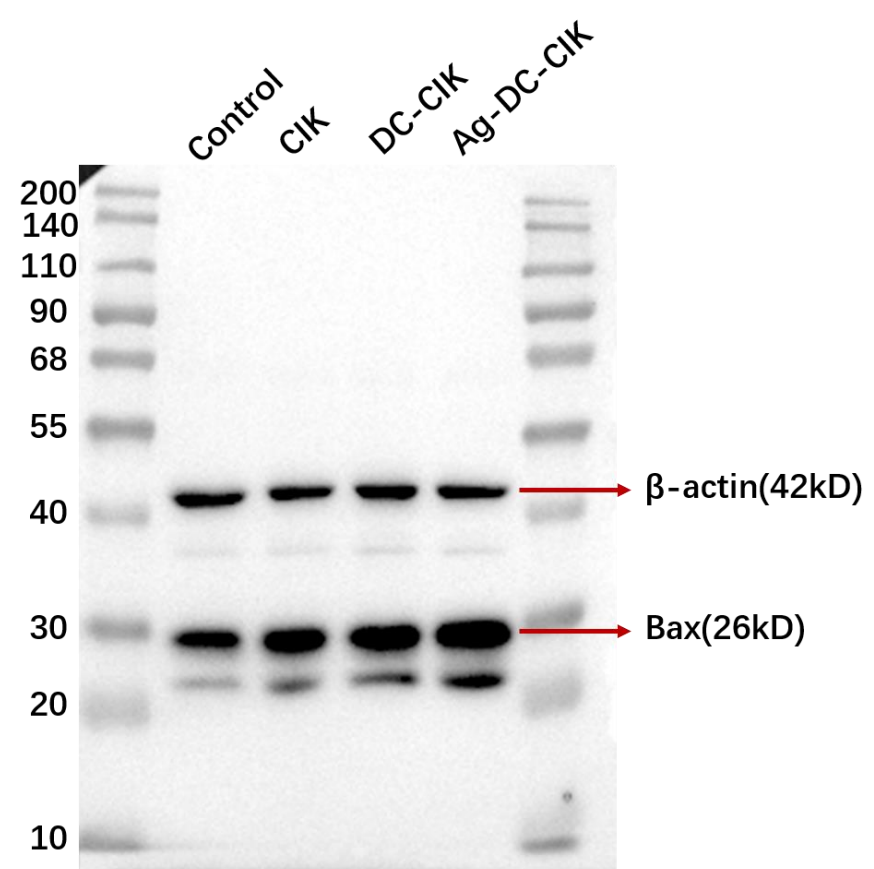

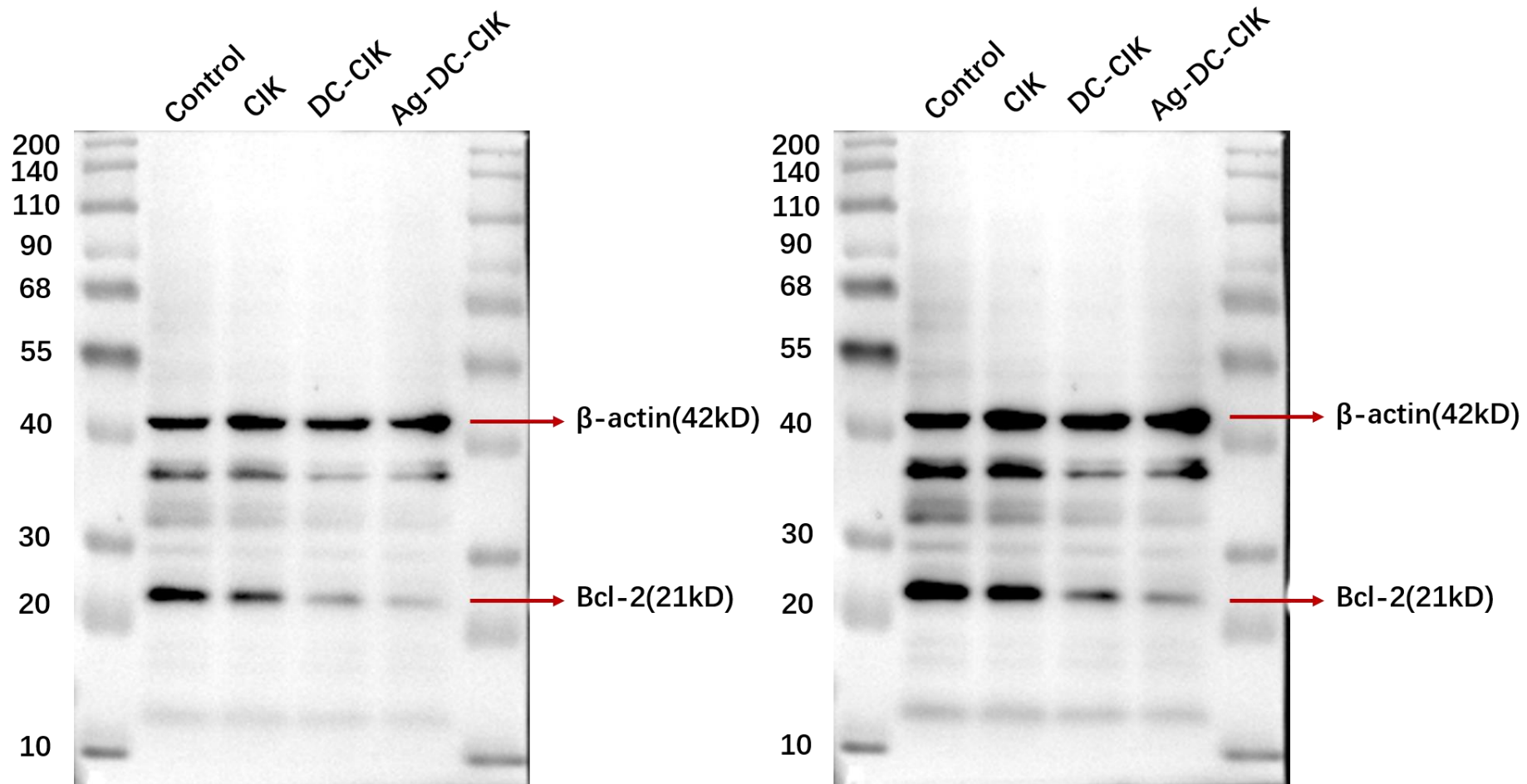

Uncropped western blots for figure S2 are found in Supplementary in Figure S4

Supplement: Supplementary file 1 [file biomolecules-15-01356-s001.zip › Figure S4.pdf]
